# Group A Streptococcus Infections: Their Mechanisms, Epidemiology, and Current Scope of Vaccines

**DOI:** 10.7759/cureus.33146

**Published:** 2022-12-30

**Authors:** Vinayak Iyer, Vivek Sagar, Devinder Toor, Valarie Lyngdoh, Gloria Nongrum, Manish Kapoor, Anuradha Chakraborti

**Affiliations:** 1 Department of Experimental Medicine and Biotechnology, Postgraduate Institute of Medical Education and Research, Chandigarh, IND; 2 Department of Community Medicine and School of Public Health, Postgraduate Institute of Medical Education and Research, Chandigarh, IND; 3 Amity Institute of Virology and Immunology, Amity University Uttar Pradesh (AUUP), Noida, IND; 4 Department of Microbiology, North Eastern Indira Gandhi Regional Institute of Health and Medical Sciences, Shillong, IND; 5 Department of Cardiology, North Eastern Indira Gandhi Regional Institute of Health and Medical Sciences, Shillong, IND

**Keywords:** streptococcus pyogenes, rheumatic heart disease, vaccine, emm gene, group a streptococcus

## Abstract

Group A streptococci (GAS) are gram-positive, cocci-shaped bacteria that cause a wide variety of infections and are a cause of significant health burden, particularly in lower- and middle-income nations. The GAS genome contains a number of virulence factors such as the M-protein, hyaluronic acid, C5a peptidase, etc. Despite its significant health burden across the globe, a proper vaccine against GAS infections is not yet available. Various candidates for an effective GAS vaccine are currently being researched. These are based on various parts of the streptococcal genome. These include candidates based on the N-terminal region of the M protein, the conserved C-terminal region of the M protein, and other parts of the streptococcal genome. The development of a vaccine against GAS infections is hampered by certain challenges, such as extensive genetic heterogeneity and high protein sequence variation. This review paper sheds light on the various virulence factors of GAS, their epidemiology, the different vaccine candidates currently being researched, and the challenges associated with M-protein and non-M-protein-based vaccines. This review also sheds light on the current scenario regarding the status of vaccine development against GAS-related infections.

## Introduction and background

Streptococci

Streptococci are common human pathogens, colonizing multiple parts of the human body, such as the upper respiratory tract, urethra, gastrointestinal tract, and oral cavity [1]. They can be cultured on blood agar plates and are classified on the basis of the observed hemolysis pattern as α, β, or γ streptococci. β-hemolytic streptococci are further classified based on differences in the carbohydrate composition of the cell wall of the bacteria. This form of grouping is known as Lancefield grouping (named after its discoverer, Rebecca Lancefield), and it consists of 20 groups labeled from A to V (excluding I and J). A majority of the pathogenic streptococci belong to group A (GAS), and the most prevalent in this group is *Streptococcus pyogenes*. GAS usually infects children in the age group of 5-15 years [1]. Group A streptococci are one of the leading causes of infectious disease-related deaths worldwide, causing nearly 500,000 deaths every year worldwide [2]. They are found to be more prevalent in the lesser economically developed nations. These bacteria were described by Theodor Billroth in 1874. He named these organisms, which he observed in cases of erysipelas and infected wounds, streptococcus, which, when translated from Greek means berry chains. Louis Pasteur elaborated on the genus when he isolated the organism from the uterus and blood of women [3].

When comparing the prevalence rates of the different groups of streptococci, particularly β-hemolytic streptococci, it was observed that group A streptococci were the most prevalent form of the bacteria in India [4]. In India, the prevalence of rheumatic heart disease (RHD) and other GAS-related infections is around 1.1 in 1000 people [5]. 

The Streptococcus cell wall contains various antigenic proteins, the most widely studied of which is the M protein [6]. This protein plays an important role in the bacteria evading phagocytosis and is encoded by the emm gene. The basic structure of the M protein consists of a conserved signal peptide, a hypervariable N-terminus, and a conserved C-terminus. The N-terminal region of this protein exhibits hypervariability, which results in the occurrence of nearly 250 different serotypes observed in GAS [7,8]. Many vaccines based on this region of the M protein have been developed due to their good immunogenicity, high specificity, and low risk of adverse reactions. Many of the candidate vaccines currently in the advanced stages of clinical trials are based on this region. These vaccines are mainly constructed using N terminals from different M proteins fused together. Recently, a novel tool for vaccine development based on the sequence similarity of different M proteins was introduced, which is known as emm clustering [9]. It was observed that certain emm-type clusters infected specific organs in the body [10]. Other vaccine candidates currently under consideration include those based on the conserved regions of the genome, such as the C-terminal of the M-protein and other similar regions of the genome. These candidates were initially found to elicit a poor immune response and be poorly immunogenic in nature, as they produced a poor immune response and caused a lot of adverse reactions in humans. However, recent advances in vaccine research and nanomedicine have helped overcome these issues [11].

There are currently no vaccines against group A streptococci in India. The current M-protein-based multivalent vaccines that have been developed show poor coverage of emm-type strains indigenous to India [12].

This has necessitated the discovery of a vaccine more suited to the Indian population and one that shows more coverage of the GAS strains indigenous to the regions. This is being accomplished through various genetic and serotype-based studies.

This review encapsulates the prevalence of group A streptococcus in different regions of India. The paper then sheds light on the status of vaccine development around the world and then compares this to the status of vaccine development in India.

## Review

Virulence factors

Group A streptococci have a variety of virulence factors encoded in their genome sequence [13]. Many of these can potentially be used for therapeutic and vaccine-related purposes. These include a hyaluronic acid capsule, M protein, the emm gene superfamily, erythrogenic toxins, and streptokinase among others. From these, the most crucial one is the M protein, as it is responsible for the majority of GAS-related infections. We will be focusing more on the M protein for the purposes of this review.

M-Protein

**Figure 1:**
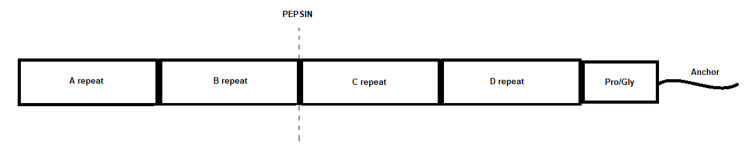
Basic structure of M-protein. The site of pepsin cleavage is indicated and occurs usually after the 228th amino acid. Note: This image is the author's own creation.

The streptococcal M-protein is one of the most widely studied virulence factors of Streptococcus bacteria. It is 490 amino acids in length on average. It is found on the cell surface and was first studied by Rebecca Lancefield in 1933 [14]. The average molecular weight of the protein is between 41 kDa and 80 kDa. It exists as an α-helical coiled dimer that extends from the cellular surface [15]. There are nearly 250 different types of M proteins. This can be mainly attributed to the variation in the number of repeats in the N-terminal region and the central domain. Despite this significant variation, all M proteins do share some basic structural components: an N-terminus that shows hypervariability, a central domain, and a conserved C-terminus. These M proteins are encoded by the emm gene. This protein can be extracted using a variety of methods, like pepsin digestion (at pH 5.8, which results in a product known as PepM) and by the use of non-ionic detergents. In both cases, the extracted M protein shows significant antigenic activity [7,13] (Figure 1). 

M proteins can be further classified based on their reactivity with certain antibodies that act specifically on the C-terminal repeats. Those that react with these antibodies are grouped under class I, and those M proteins that do not react are grouped under class II. Class II strains contain a factor known as serum opacity factor (SOF) and are designated as SOF+/ OF+, while the class I strains lack SOF and are labeled SOF-/OF-. Their main role in pathogenesis is the inhibition of phagocytosis. This is carried out by binding of the protein to complement inhibitory regulators like factor H, C4 protein, and also with fibrinogen, which as discussed earlier, blocks the interaction of the C3b molecule and stops the complement cascade [16]. Streptococcal M protein is also capable of generating autoantibodies leading to autoimmune diseases like acute rheumatic fever (ARF). Kaplan and his colleagues first published their findings that demonstrated the presence of immune cells like gamma globulins against the heart tissues found in patients with streptococcal infections deposited on the sarcolemma of the tissues [17]. They also observed various levels of damage to the tissues. For instance, damage to the sodium and potassium channels, which are critical to the normal functioning of the cardiac tissues, was observed. This could be due to the continued action of antibodies that are present in these tissues. Streptococcal infections can lead to autoimmune disease through various means, such as the targeting of cytoskeletal proteins that share structural homology with the M protein. This was demonstrated by a study that showed that the proteins implicated as antigens in an autoimmune reaction include host cytoskeletal proteins like laminin, which is composed of three alpha-helical chains. This protein is structurally very homologous to the M protein alpha helix and is sometimes recognized by the host anti-streptococcal antibodies [18]. Another study focused on a specific epitope present in the N-terminal region of the M5 protein called NT4 (which is a T-cell epitope). This study found extreme sequence homology between human cardiac myosin and specific fragments in the M5 and M6 proteins [19]. This suggested that in people suffering from RHD, T cells that are cytotoxic for the cardiac muscles are produced, which leads to the degeneration of the targeted tissue, which in this case is the mitral valve. This sharing of epitopes between host antigens and the pathogenic organism that occurs due to either identical amino acid sequences, homologous amino acid sequences, or the presence of epitopes in different types of molecules is termed as molecular mimicry.

Emm Gene

The streptococcal virulence factor M protein is encoded by the emm gene. It is part of a wider array of related genes, which include certain immunoglobulin-binding proteins and the M proteins (also known as the emm gene superfamily). A group of related genes that show sequence similarities or are under common regulatory control can be termed as a gene family. A gene superfamily, however, has more number of genes that are similar in characteristics like domain structures, nucleotide or protein sequences, etc. They can show different expression patterns [20]. Members of the emm family of genes include emm, Mrp, and enn. One of the main regulators of M protein genes is mga [21]. The mga regulator is found in all GAS strains and has two allelic forms, mga-1 and mga-2. This regulatory gene is responsible for the activation of the M protein and a host of other streptococcal virulence factors. These virulence factors are important for adherence to host tissues and for avoiding host immune surveillance, among other things [22]. The mrp gene is similar to the emm proteins, but they lack the C-terminal repeats. Emm proteins work by binding to complement regulators like C4 binding protein (C4BP).

There are various methods utilized by the emm gene and its related proteins. The importance of M proteins in evading phagocytosis has been debated. Some studies have shown that certain strains are able to resist phagocytosis even in the absence of M protein [22]. This is especially seen in SOF+ strains, where the Mrp and Enn proteins bind to the host antibodies and resist phagocytosis. Emm proteins also bind to IgA via a short stretch of residues near the N-terminus (as seen in the emm4 protein). Cell adhesion is another important process by which streptococci can cause infections. This is mediated by proteins such as adhesins and can be highly cell-specific (as demonstrated by Courtney et al.) [23]. Courtney and his colleagues, in their review [24], described a widely accepted model by which streptococci adhere to host tissues. The first step involves a weak interaction of lipoteichoic acid (LTA) with fibronectin present in host cells. This is followed by the binding of various M- and M-related proteins that are specific to the type of target tissue. The specificity of the cell type to which a specific strain of Streptococcus binds also depends on the M protein serotype. Different emm genes encode different M-proteins. These proteins, in turn, bind to different ligands in the host tissue. For instance, the M3 protein was shown to bind to specific types of collagen due to the presence of a collagen-binding region in its N-terminal. Tissue tropism to Hep-2 cells was observed in M24 cells [25]. Other proteins play an important role in the adherence and colonization of the bacteria on host tissue. These include fibronectin-binding proteins (e.g., SfbI), glyceraldehyde 3-phosphate dehydrogenase (GAPDH), and α-enolase.

Other Virulence Factors

Besides the M protein, there are many other virulence factors present in the streptococcal genome.

Hyaluronic acid: This is a polymer composed of N-acetylglucosamine and D-glucuronic acid. It is encoded by the hasA, hasB, and hasC genes. It plays a key role in evading phagocytosis. One of the ways in which this is accomplished is due to the structural homology shared between bacterial hyaluronic acid and its human form [26,27]. Some serotypes, like the M4 and M22, lack this capsule. These isolates, however, do possess the hyaluronate lyase enzyme, which degrades the hyaluronic acid [28]. This enzyme is usually inactive in many other strains due to a point mutation in its encoding sequence. Starr and Engleberg, in their paper [27], hypothesized that strains that lack the capsule utilize the hyaluronidase enzyme to degrade the hyaluronic acid in the host cell extracellular matrix (ECM). They also proved that GAS can also utilize the breakdown of hyaluronic acid as a potential carbon source, especially in nutrient-starved conditions.

Streptokinase and plasminogen binding protein: Streptokinase is a 414-amino acid long protein involved in the conversion of inactive plasminogen to its active form-plasmin. Streptokinase was one of the first genes to be sequenced from group C streptococcus in 1984 [29]. The gene encoding for this streptokinase was labeled as skc and cloned from the H46A strain of *S. equisimilis *using λ vector and *E coli* plasmid. In 1989 [30], scientists were able to clone the streptokinase gene ska from the M49 strain of GAS. This was found to be 90% homologous to the skc gene. The streptokinase gene structurally comprises the α, β, and γ domains. The α and γ show a significant level of conservation among the different strains, whereas the β domain shows sequence variability. The streptokinase protein secreted by the bacteria acts on the host plasminogen to convert it to plasmin and activates various metalloproteinases that degrade the ECM and basement membrane and aid in the migration of the bacterium into the cells [31,32]. The plasmin protein is encoded by the PLG gene and is involved in the dissolution of plasma proteins and fibrin clots [33]. The streptococcal plasminogen and the streptokinase protein help in disrupting the host’s extracellular matrix, and after colonization, the plasmin protein disrupts the host’s complement pathway by cleaving C3b and C5 proteins [34,35].

C5a peptidase: Streptococcal C5a Peptidase (SCPA) is a 130 kDa serine peptidase. Its main function is the inactivation of the complement pathway by cleaving the C5a protein and inhibiting its downstream action and phagocytosis [13]. This enzyme has also been implicated in the cleavage of the C3 complement protein. It also inactivates the C3a protein. In the context of GAS pathogenesis, it was recently discovered that the enzyme plays an important role in the adhesion of the bacteria to the host endothelial and epithelial cells [36].

Pilus protein: Pili are hair-like structures present in bacterial cells. They play a variety of roles, including adhesion, biofilm formation, etc. In *S. pyogenes*, pilus proteins are encoded by the fibronectin and collagen-binding protein T-antigen island (FCT) [37]. Nine such islands have been discovered in the streptococcal genome. They play a vital role in the adhesion of the bacteria to the host pharyngeal cells. Pilus proteins are structurally composed of repeats of backbone protein (BP1), ancillary protein (AP1), and ancillary protein 2. Deletions of these specific subunits and also of the specific FCT islands have been shown to decrease bacterial survival [38].

Understanding the various virulence mechanisms of streptococci will help us better understand its prevalence, and what makes people susceptible to it, and also improve vaccine development.

Epidemiology

Group A streptococci are one of the leading causes of infectious disease-related deaths worldwide. They are found to be more prevalent in the lesser economically developed nations. The advent of antibiotics (like penicillin) briefly helped control the spread of GAS infections. However, in recent years, a lack of uniform data regarding the surveillance of GAS-related diseases brought about the need for uniform surveillance methods of these diseases in order to keep track of them and facilitate research into them.

Global Scenario

There have only been a few studies documenting the global impact of streptococcal diseases. However, there have been many country-wise studies that have been carried out for the same. A study carried out by Carapetis and his colleagues [2] attempted to study the impact of streptococcal diseases, particularly rheumatic fever, on the global population. They compiled results from various countries (that were grouped regionally and then also categorized on the basis of economic development), and data pertaining to the prevalence and mortality rate were presented. According to this, 1.78 million cases of severe GAS infections occur every year, and there are an estimated total of 18 million cases so far. This report was also used by the WHO for their official statistics [39]. According to this, the incidence rate of RHD was highest in the sub-Saharan African Region (six in every 1000 people). According to a study based on the indigenous population of Australia, the highest prevalence of cases was in the age group of 25-34 years, with an incidence rate of 22.1 per 1000 population [40]. Myanmar too showed a similar peak in the prevalence rate in the age group of 26-35 years (26.4 in 1000) [41]. A study carried out among schoolchildren in Nepal found the prevalence of RHD to be around 1.2 per 1000 children [42]. The WHO study revealed that nearly 80% of the global RHD cases in children aged between 5 and 14 years came from lesser developed countries. These countries also account for 80% of the global population. Correlating these numbers gives us a global figure of 16 to 18 million cases of RHD every year. The study did raise questions about some of the data from countries like the Philippines and Thailand and suspected them of under-reporting the figures. The WHO study also raised questions about various other aspects of the study, like how some of the studies were performed in urban areas instead of rural areas, which would lead to a lower prevalence being reported. It also extrapolated data from other studies regarding acute rheumatic fever (ARF), which were conducted jointly by scientists in the USA and UK, as well as from studies in Australia. This showed that ARF first occurs in children at the age of 5-14 years and that nearly 60% of all ARF patients soon develop RHD.

Rheumatic heart disease accounts for the majority of GAS cases, with 15.6 million cases globally and 282,000 cases occurring each year [39]. The mortality rate of RHD varies from country to country. Very few countries have reliable data regarding the mortality rate of RHD. Even though sub-Saharan African countries show the highest incidence rate of RHD, there is little to no data about the mortality rate of this disease. South Africa is the only African country that reports such deaths to the WHO. Kazakhstan had the highest mortality rate of five per population of 100,000. 2005 WHO report estimated that 492,000 people die due to RHD annually. This figure was arrived at by using age-adjusted rates and carefully studying the demographical differences between the indigenous and non-indigenous populations in Australia, Alaska, and New Zealand. The mortality rate from these studies was used to correlate to the global mortality rate. However, there is still a need for proper and accurate figures regarding the mortality rate of RHD, especially in less developed countries.

Another major condition caused by group A streptococcus is post-streptococcal glomerulonephritis (PSGN). Its incidence rate was found to be 472,468 cases [39]. This disease affects the kidneys and can eventually lead to renal failure (an average of 6% of glomerulonephritis patients eventually develop renal failure). Streptococcal infections also gave rise to conditions like encephalopathy and hypertension. GAS infections are one of the most common causes of bacteremia in children, with a reported prevalence of nearly 63 cases per 100,000 children aged two years and under. GAS also colonizes the birth canal, which can lead to neonatal bacteremia. This condition is especially prevalent in poorer countries like the Philippines, Ethiopia, and Papua New Guinea. One other inference from various studies regarding GAS infections is that the prevalence of these diseases can be directly correlated to the socioeconomic status of the population. Such infections are more prevalent in poorer communities [43]. One such example can be seen in New Zealand and Australia, where the prevalence of diseases like a rheumatic fever [40] was higher in the Aboriginal population when compared to the urban population.

Although few studies have been conducted since 2005, there has not been any concrete global study. All studies have been either specific to a certain region in a country or to a specific country. According to the latest Centers for Disease Control (CDC) data, nearly 24,000 cases of GAS infections are reported in the USA alone [44]. A paper published in 2018 found that the incidence rate of invasive GAS infections in Alberta, Canada, over a 14-year period from 2003 to 2017 rose from 4.24 to 10.24 per 100,000 population. The most at-risk population were at the extremes of age, with children <1 year of age having an incident rate of 9.69 and persons over 60 years having an incident rate of 11.69 [45].

**Figure 2:**
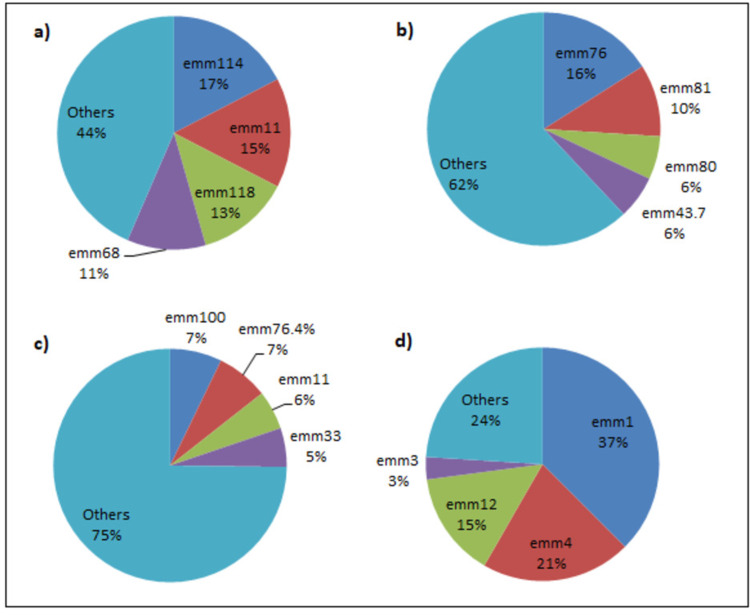
Emm type distribution in various regions around the world. (a) Bocking et al. [48] (Canada) (n= 46; where n is the total number of emm-typed isolates that were obtained). (b) Barth et al. [50] (South Africa) (n= 233). (c) Steer et al. [51] (Fiji) (Pacific region; n=55). (d) Oliver et al. [52] (Australia) (n= 96). Note: All the pie charts in the figure are the author's own creation.

Significant variation is seen in the global distribution of emm types also. A set of 25 emm types, including emm1, emm12, and emm28, accounted for 90% of all isolates from developed countries [46]. emm1 and emm12 were the most common types seen in developed nations and accounted for nearly 40% of all the isolates in Asia and Latin America. In a study by Gherardi et al. [47], in their review of the prevalent emm types in European and North American countries found emm3 and emm1 were found to be the most prevalent serotypes in countries like England, Germany, and Spain. In separate studies conducted in Ireland and Hungary, the emm1 serotype accounted for nearly half of all isolates found. Countries like the Czech Republic even reported a significant prevalence of certain uncommon serotypes like emm53 and emm81. North American countries also showed similar results, with emm1 being the predominant type. In the USA, emm1, emm3, and emm28 were the most common serotypes. This result was quite uniform across three separate studies conducted across the country. Bocking et al. [48] in their study identified 14 different emm types from 65 GAS samples collected from the indigenous communities in the Ontario region of Canada. Some of the prevalent serotypes discovered included emm114, emm118, emm87, and emm1. Instead of a single dominant emm-type, the Asian, Pacific Asian, and African countries had more than one serotype with significant prevalence. In North African countries, emm12, emm89, and emm3 were the more prevalent types, along with emm1. emm87 was found to be the predominant type in a study carried out in Yemen in 2006. Other prevalent emm types in the North African and Middle East regions include emm6, emm4, and emm18 [49]. A study conducted in South Africa found 46 different emm types, with emm76, emm81, and emm80 being the more prevalent ones [50]. A significant variation was seen in Pacific Countries like New Zealand, Australia, Fiji, etc., especially in the indigenous population. While the emm-type distribution in the samples isolated from the urban population was quite similar to the ones isolated from European and North American countries, the isolates from the indigenous population showed significant variation in the serotypes. Some of the prevalent emm types included emm54, emm76.4, emm100, and emm4 [51,52]. This trend of greater variation among indigenous populations was also observed in Canada. Steer and his colleagues carried out a study where they sequenced both the N-terminal and the C-terminal regions [53]. They were able to collect 817 isolates of GAS, group C streptococcus (GCS), and group G streptococcus (GGS). The 535 GAS isolates yielded 67 emm types and 74 emm subtypes, which are summarized in Table 1 and Figure 2. The authors were able to successfully sequence 512 of the 535 GAS isolates at the C-terminal region, specifically the J14 region. They obtained 19 different J14 types, of which J14.0 and J14.1 were the most common types observed. The study also revealed a new emm type and four new subtypes. Very few studies exist that detail the variation of serotypes in a specific region over a long period of time. One study by Shulman et al. surveyed the geographical and temporal distribution of emm-types in the USA and Canada [54]. Significant variation was observed in the prevalence of serotypes in each region over the course of the study.

**Table 1:** Summary of the prevalent emm types in different regions of the world.

Region	Prevalent emm type	Author
Europe	emm1, emm3, emm12	Gherardi et al. [47]
North America	emm1, emm3, emm28, emm114, emm118, emm87	Gherardi et al. (USA) [47], Bocking et al. (Canada) [48]
North Africa and Middle East	emm12, emm89, emm3	Rafei et al. [49]
South Africa	emm76, emm81, emm80	Barth et al. [50]
Pacific	emm76.4, emm100, emm106, emm54, emm55, emm42 emm92, emm70, emm76.4	Steer et al. (Fiji) [51], Oliver et al. (Australia) [52], Steer et al. (Fiji) [53]

Indian Scenario

The actual impact of GAS on the Indian population and the various factors behind it are still poorly understood. In India, it mostly affects children between 5 and 15 years of age. The incidence rate of GAS infections and their related diseases has shown some variation in their prevalence depending on the region. A review by Brahmadathan [55] stated that the prevalence rate of RHD was between 1 and 11 per 1000 children in India. Though GAS infections have shown a significant decline in their prevalence, they are still a sizeable burden on the population of some of the less developed countries. Post-streptococcal glomerulonephritis, a kidney disease that occurs as a result of streptococcal infections, has a prevalence rate of only 0.3 cases per 100,000 people. But in less developed countries, it shows a significantly higher incidence rate. For instance, in India, the prevalence rate of post-streptococcal glomerulonephritis is estimated to be around 39.24 per 100,000 persons [56].

The exact impact of RHD and other GAS-related diseases is still poorly understood. Most of the studies just provide an estimate of the number of cases by extrapolating the figures from their surveys to the general population. A hospital-based surveillance program was initiated in 2000 by the Indian Council of Medical Research (ICMR) for rheumatic fever (RF) and RHD. According to this survey, the prevalence of the two diseases across the country was estimated to be around 1.2 per 1000 people [5,57]. 

**Figure 3:**
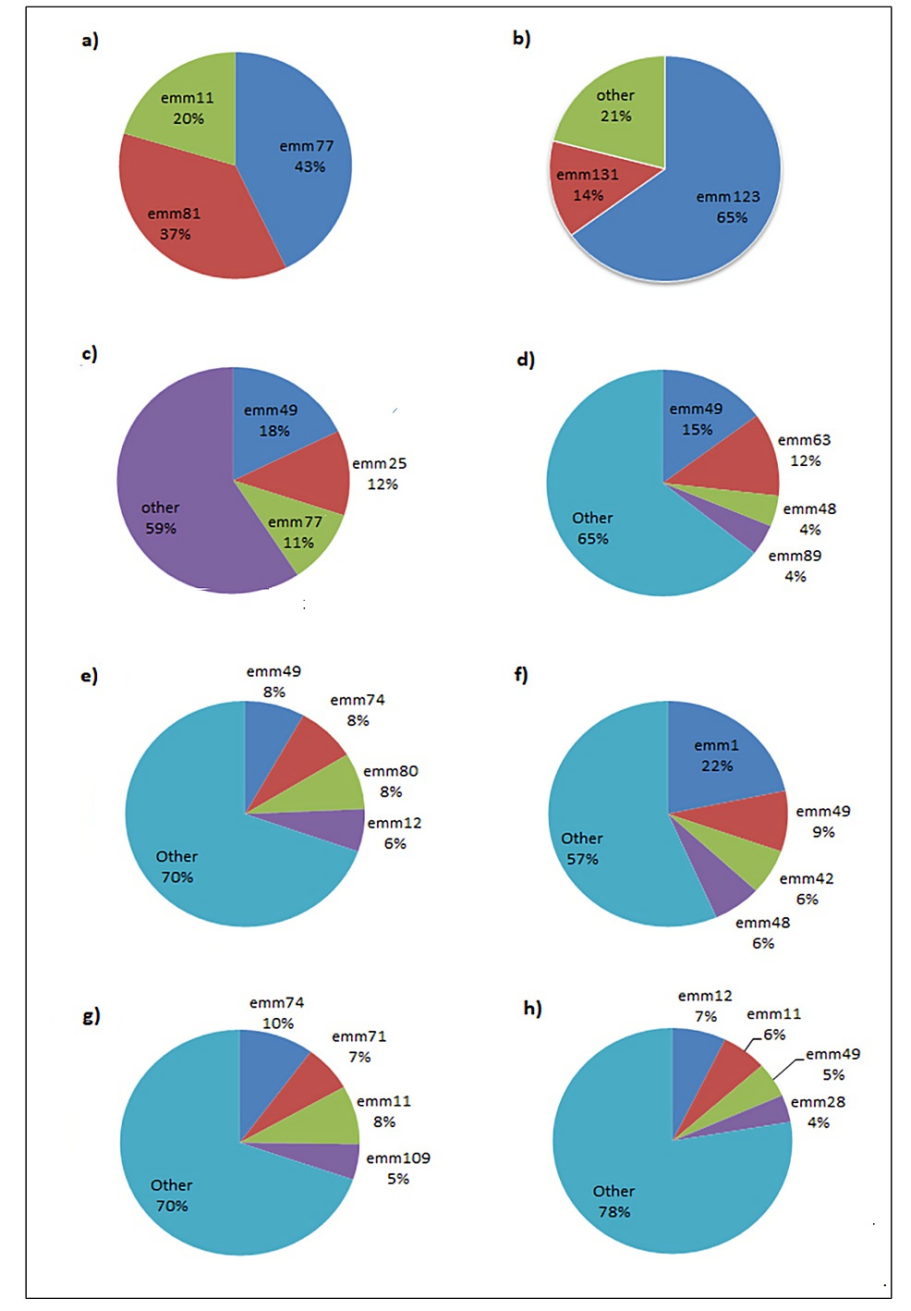
Emm type distribution in various regions of India. (a) Dhanda et al. [58] (North India) (n=71; where n is the total number of GAS isolates that could be characterized for their emm type). (b) Devi et al. [59] (East India) (n=14). (c) Ray et al. [60] (East) (n=140). (d) Balaji et al. [61] (South) (n=67). (e) Haggar et al. [62] (North and South (cohort study)) (n=49). (f) Arya et al. [69] (North and West) (n=92). (g) Dey et al. [64] (North) (n=59). (h) Sagar et al. [66] (North and South) (n=201). Note: All the pie charts in the figure are the author's own creation.

There is significant variation seen in the diversity of emm strains in the different regions of India. A study was carried out by Dhanda et al. to study the virulence factors in GAS isolates in north India. The prevalent emm types in the study were emm74, emm11, and emm68 [58]. A small-scale study carried out in Assam found emm123 as the most prevalent type in the Eastern Indian population [59]. A larger study was carried out in Tripura, where samples were collected from various tertiary care hospitals in Kolkata and certain other towns. In all, 270 samples were collected, of which 140 were GAS isolates. Emm typing was carried out, and emm49, emm77, and emm25 were observed as the more prevalent types [60]. A study by Balaji et al. found 67 *S. pyogenes* isolates from 370 throat swabs in southern India. Emm49, emm63, and emm100 were found to be the prevalent serotypes [61]. Another collaborative study between institutes in North and South India showed great heterogeneity in the emm types. Certain common subtypes were emm49, emm74, and emm12. However, certain subtypes were region-specific (like emm74 and emm80) [62]. A study by Menon et al. [63] was one of the first surveys carried out in southern India regarding the serotype diversity of *S. pyogenes*. It was a small-scale study, with only 34 isolates being collected. Eleven different emm types were identified from this. Emm92 was found to be the prevalent strain. On comparing the serotypes identified in this study with the common emm-types in the USA, it was found that only emm12 was the common serotype between the two populations. Another study by Dey et al. [64] demonstrated the extensive variability of emm-types in India. The study was carried out in north India and identified 33 different serotypes among 59 pharyngitis isolates. One important point to note from the papers regarding the prevalence of the bacteria in north India is the presence of certain subtypes [65], like the emm1-2 serotype [55]. While previously considered a subtype of emm1, detailed sequence comparison revealed that the emm1-2 strains share only 90% homology with the emm1 strain (for a strain to be considered a subtype, it needs to share 95% sequence similarity with the parent strain). This difference was further elaborated upon, and new distinct strains were found that were originally thought to be the emm1 serotype, one of them being the emm1-2 strain [66,67] (Table 2, Figure 3). A few studies have been carried out in North India since 2000 that map the serotype distribution of GAS. Although some serotypes were consistently observed in the studies (like emm81, emm112, and emm11), high diversity in serotypes along with the emergence of certain subtypes like emm81.1 and emm112.2 were also observed [68]. Epidemiological studies and emm type distribution are summarized in Table 2 and Figure 3, which illustrate the variation observed across the different regions of India. This can be attributed to various factors, such as differences in population genetics and environmental differences found in the respective regions.

**Table 2:** Summary of epidemiological studies on group A streptococcus in India.

Region	Prevalent emm type	Author
North	emm74, emm11, emm68	Dhanda et al. [58]
East	emm123	Devi et al. [59]
East	emm49, emm77, emm25	Ray et al. [60]
South	emm49, emm63, emm100	Balaji et al. [61]
North and South (cohort)	emm12, emm28, emm49 (south), emm74, emm80, emm1-2 (North)	Haggar et al. [62]
North	emm74, emm71, emm11	Dey et al. [64]
North and South	emm4, emm11, emm12, emm3, emm28 (south), emm2, emm22	Sagar et al. [66]
North and West	emm1, emm49, emm42	Arya et al. [69]

Owing to such variations in emm serotypes and the high prevalence of RHD and other GAS-related infections, it is imperative that a vaccine must be introduced to reduce the mortality rate and the occurrence of such infections. Certain factors from the streptococcal genome have been identified as potential vaccine candidates. Some of them have been summarized below.

Streptococcus vaccines

Introduction and History

The high prevalence rate of streptococcal diseases, especially in lower-income countries, has necessitated the need for the development of a new vaccine. Research into vaccines against streptococcal infections has been going on for many years now, but there are still quite a few hurdles to cross before an effective vaccine that can help us successfully eliminate this infection is developed. Young children are the worst-affected subset of the population in any country. They show a higher prevalence of infection when compared to adults. One reason behind this could be the acquisition of type-specific immunity that occurs after an infection. Antibodies are generated against the specific M proteins after the infection, which persist for many years after it [70]. This persistence of antibodies was first demonstrated by Rebecca Lancefield in 1959 [71]. The first attempt at a vaccine was by Young and his colleagues [72]. They used heat to kill whole cell extracts, which failed to induce any immune response. Soon, specific crude extracts of specific proteins were used as potential vaccine candidates [73]. Fox and his colleagues were the first to demonstrate the use of purified M protein as a vaccine candidate against streptococcal infections [74]. They used a purified form of the M protein isolated from GAS as the vaccine candidate, and the subjects were injected with a virulent strain of type I streptococcus 30-50 days after the administration of the vaccine dose. The volunteers did not show any sign of infection. This demonstrated the presence of acquired immunity. Another unique part of the studies conducted by Fox and his colleagues was that they were the first to use a purified form of the protein. Earlier attempts at developing a vaccine were not successful as they also resulted in the development of many side effects due to the crude extracts of the candidate molecule used and the presence of other contaminants and toxins (like pyrogenic toxins). Though these early studies were successful in demonstrating persistent, acquired immunity through the use of vaccination, they still suffered from a few drawbacks. The major drawback was that the immunity acquired was highly type-specific. The host was immune only to a certain M protein that was present in the symptomatic infection. There are more than 250 different types of M proteins that exist. Even during the time of Lancefield’s research, there were about 80 different types of M proteins. Therefore, it is necessary to develop a vaccine that can act against multiple M serotypes in order to grant sufficient immunity to the host. Another concern that has been raised is that some of the antigens that are used in vaccines could contain epitopes that trigger autoimmune reactions. In the case of streptococcal vaccines, this could lead to conditions like acute rheumatic fever (ARF), which is something the vaccine was originally designed to prevent. The Streptococcus genome and the cell itself contain many potential target candidates for vaccine development. These include M proteins, actin-binding proteins, genes encoding the pilus of streptococci, the C-repeat region of the M protein, etc. Currently, there are a few vaccines that are under development around the world. The current vaccine situation is summarized below.

Global Scenario

The resurgence of invasive pathogens like streptococci in the past 30 years has pushed the focus of research into finding an efficient vaccine for these diseases. While many candidate molecules for streptococcal vaccines have been discovered, none are as yet available in the market as they haven’t cleared the necessary trials.

The type of vaccine being researched for Streptococcus can be classified on the basis of the target region of the bacterial cell. Fox and his colleagues were the first to use a purified form of a protein (M protein) as the candidate [74]. They used alum as an adjuvant in the vaccine. Since then, many more target molecules have been discovered as vaccine candidates. These can be broadly classified as M-protein-based vaccines and non-M-protein vaccines. M-protein-based vaccines can be further classified based on whether the target region is the N-terminal or the C-terminal of the M-protein.

M-protein-based vaccines are one of the most widely studied proteins in the Streptococcus genome. There are nearly 250 different serotypes of the M protein, and this variation in the serotype has been the focus of a lot of research on Streptococcus bacteria, both regarding its role in pathogenesis and as a potential vaccine. This protein was first studied extensively by Rebecca Lancefield in 1932 [14]. In a subsequent review in 1962, she described its role as a major virulence factor [71].

In N-terminal, early attempts at a potential vaccine for streptococcal vaccines made use of a heat-killed, whole-cell extract of Streptococcal M protein [72]. These preparations were extremely crude in nature and showed a high level of reactogenicity. This approach was then narrowed down to using certain proteins from the genome. Early M-protein-based vaccines used an acid extract of whole M proteins [73]. These also suffered from similar problems, and the final dose amount that was delivered was too small to elicit a significant immune response. Fox and his colleagues in 1966 [74] were the first to use a purified form of M protein to induce a long-term immune reaction in test subjects. Subsequent studies were carried out using specific M serotypes, most notably in 1979 by Beachey and colleagues, who used a pepsinized extract of the M24 protein and were able to successfully show type-specific immunity in the volunteers [75]. These initial studies were extremely specific to the M-protein serotype used, and hence the person could potentially still get sick if infected with different M-type streptococci. Beachey and his colleagues then used a hybrid form of the N-terminal from the M5 and M24 proteins. This was the first time that something other than a monovalent form of the M-protein was used as a vaccine candidate. When injected into a rabbit, antibodies were raised against both M5 and M24 streptococci [76]. This was then expanded step by step to include a greater number of serotypes, a tetravalent form of the protein [77], and a hexavalent form [78]. The hexavalent form used M3, M1, M6, M19, M5, and M24 serotypes, and 28 volunteers were used. This hexavalent peptide was able to significantly induce antibodies against the different serotypes. A phase 1 trial of this was conducted, along with a one-year follow-up of the patients who were enrolled in the study. The vaccine was found to induce a significant level of antibodies, and no cross-reaction was observed [79]. Hu and her colleagues in 2002 came up with a 26-valent streptococcus vaccine [80]. They constructed this vaccine using the amino terminals from 26 different M serotypes. The M types were selected based on epidemiological data from North America and Europe of invasive streptococcal strains, especially those which were implicated in causing ARF and RHD (like M19 and M24). Its safety and immunogenicity were also tested in a clinical trial consisting of 30 volunteers [81]. The vaccine induced a nearly fourfold increase in antibody titer against nearly all of the 26 serotypes used in it. This vaccine was further improved by including four more M proteins in order to grant more coverage to the vaccine. The strains used in this 30-valent vaccine [82] accounted for 98% of the pharyngitis cases in the USA and Canada and also covered 78% of the invasive diseases in Europe. Immunogenicity was tested in rabbits, and it was found that an 800-microgram dose induced a significant immune response. No cross-reaction against human tissues was observed during the experiments. This vaccine has undergone a randomized phase 1 study in Canada and USA and has shown favorable results [83]. There have, however, been some concerns regarding this vaccine. Though the serotypes used in this vaccine covered nearly all the serotypes in North America, they showed poor coverage of the different serotypes in indigenous populations and those in the poorer, developing nations. A study by Giffard [84] and his colleagues on the efficacy of the vaccine on the aboriginal population of Australia showed that it only covered only 40% of the emm types associated with throat infections in the region and 25% of the serotypes associated with soft tissue infections. More importantly, it did not include emm55, which is a common serotype associated with acute post-streptococcal glomerulonephritis. Another paper by Abraham [12] showed that this vaccine only covered 22 out of the 135 emm types surveyed across India.

**Figure 4:**
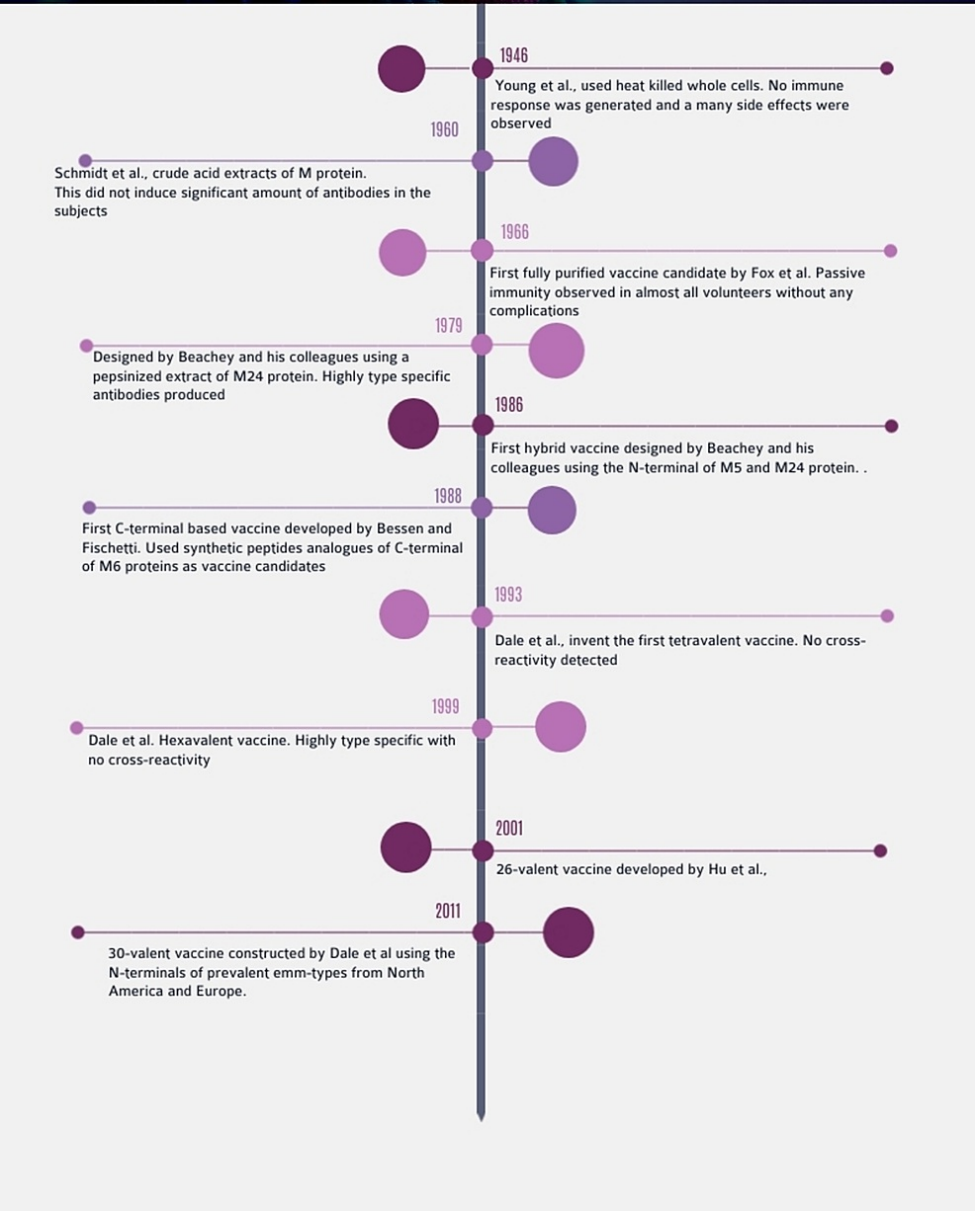
Timeline of M-protein-based vaccines (from 1946 to 2011). Note: This image is the author's own creation.

In C-terminal, the above-mentioned vaccines focused on the variable N-terminal region, studies have also found certain peptides in the conserved C-region of the M-protein. An example of this is the B-cell epitope, J8. Various studies have been carried out that show that it can be used as a vaccine candidate. Olive et al. were able to prove the immunogenic and opsonization capacities of the J8 peptide attached to a synthetic lipidic polylysine core peptide (LCP) system (which acts as an adjuvant) [85]. This LCP-J8 system was able to elicit a strong IgG response in B10.BR mice. This system showed significant opsonization activity against certain M strains of GAS, especially when coupled with complete Freund’s adjuvant (70% opsonization activity). Batzloff and his colleagues conjugated the peptide with the diphtheria toxoid (DT) protein in order to increase its immunogenicity and then tested using various adjuvants (SBAS1 and SBAS2) [86]. SBAS2-adjuvanted J8 vaccine was able to induce a higher IgG response compared to SBAS1 and J8 conjugate with DT alone. A phase 1 trial was conducted with 10 participants (including two placebo subjects) using the J8 vaccine candidate conjugated to DT protein, and alum was used as an adjuvant [87]. Significant increase in serum antibody levels was observed after 28 days. The study did emphasize the need for multiple doses to be administered over time as the antibody levels dropped to baseline levels after six months. Mills et al. [88] later developed a high-density microarray patch (HD-MAP) onto which the vaccine could be adjuvanted. This patch is placed on the skin, and the vaccine candidate enters the dermal layer slowly. They found that this method induced a better, more accelerated IgG response when compared to the normal alum adjuvant method. Another vaccine targeting a conserved C-region of the M protein was developed by Guilherme and their team [89]. This vaccine candidate is based on a 55 amino acid residue in the C-terminal of the M5 protein and covers the B-cell and T-cell epitopes of the protein structure. This candidate exhibited a significant capacity to elicit a sustained immune response without any cross-reactivity when tested in Balb/c mice. This vaccine has only undergone testing in non-rodent species (minipigs) for evaluating safety so far, but it has shown promising results in them [90]. A brief timeline of the M-protein-based vaccines has been shown in Figure 4.

In non-M-protein-based vaccines, scientists have also been studying other components of the Streptococcus genome in order to find a more universal vaccine candidate. The above-mentioned M-protein-based vaccines show significant variation in their N-terminal sequences, and this makes it difficult to come up with a suitable vaccine for the different populations around the world. This has led scientists to look at other components in the Streptococcus genome in their hunt for a novel vaccine candidate. Some of these have been discussed. 

Cysteine proteases are a class of proteins that have been implicated in the degradation of ECM proteins. These enzymes were first investigated as potential vaccine candidates by Kapur et al. [91], who used a denatured form of the proteins. They observed a significantly higher level of passive immunity against highly virulent strains of *S. pyogenes*.

The fibronectin family of proteins is involved in bacterial cell adherence and colonization. Some members of this family include Sfb1, SbX, and protein F. Sfb1 is a protein involved in the adhesion of the bacteria to the host cells. Schulze and his colleagues [92] used a recombinant form of the fibronectin-binding domain of Sfb1 and were able to induce IgG and IgA responses against *S. pyogenes* in mice. This protein, however, was not found to be effective in the case of systemic infections [93]. Schulze, along with Olive and his team, developed a vaccine that was composed of a 148-amino acid-long minimal fibronectin-binding repeat (FNBR) domain and the J8 epitope of M-protein. The FNBR sequence is present in the binding domain of Sfb1. This was conjugated to a lipid core peptide (LCP) and co-administered with a TLR2/6 agonist. They found that this combination was able to confer protective immunity in mice even against a lethal dose of *S. pyogenes* strain N192 [94]. A 2005 study by Kawabata et al. [95] used a highly conserved protein known as FBP54. They carried out nasal and oral immunization of mice using this protein and found that this induced a significant IgG and IgA immune response. The conserved nature of this protein (98%) makes it a promising vaccine candidate. Further studies on *in vitro *and *in vivo *studies on fibronectin proteins as vaccine candidates are still ongoing.

Emm clustering is a new tool being developed in vaccine research and is the method of clustering related closely related emm-types into clusters [9]. This concept was first elaborated by Sanderson-Smith and her colleagues. They were able to group 175 emm-types into 48 different clusters, which were divided into two clades. emm-types within a single cluster exhibited nearly 70% pairwise identity. Closely related clusters can induce cross-opsonization and an increase in the antibody titer. This was especially evident in the E4 and E6 clusters [96]. This cluster typing method can be used as additional information on epidemiological data and also help us understand the functional classification of the different emm types better. For instance, Cluster pattern E corresponds to the SOF+ class, while the patterns A-C and D correspond to the SOF- class. This method of clustering different M-types can greatly improve our understanding of the different serotypes and also provide a base for improvements in vaccine research. This method of emm-clustering was used to design a vaccine that was effective against emm types from a particular cluster. Dale and his colleagues [97] identified the E4 cluster, which contains 17 serotypes that are very common in the USA and were used in the study. Five N-terminal peptide sequences spanning the 17 emm types were combined to create a new vaccine candidate, and its immunogenicity was evaluated on rabbits. Cross-reaction of the rabbit antisera was observed against all five peptide sequences, and emm type-specific antibodies were generated against 15 out of the 17 emm types in the E4 cluster.

Vaccine based on carbohydrates: Sabharwal and his colleagues [98] were able to successfully confer protection against GAS by using GAS-specific carbohydrates from an M-negative strain. No cross-reaction was observed against human tissues. A multicomponent vaccine comprising five common GAS antigens was developed (the Combo5 vaccine) by Rivera-Hernandez and her team, which had previously been shown to confer protection in murine models [99]. This vaccine was composed of five GAS antigens, namely arginine deiminase (ADI), trigger factor (TF), C5a peptidase (SCPA), an IL-8 protease (SpyCEP), and streptolysin O (SLO). Certain components, like N-acetyl glucosamine (GlcNAc), were not included as they have been linked to the production of autoimmune reactions. This vaccine candidate was tested on BALB/c and AlbPLG1 mice and was able to induce a significant antibody response against each of the antigens. They further tested this on rhesus monkeys (as a non-human primate study) [100]. A significant reduction in the severity of pharyngitis was observed in the monkeys treated with this vaccine.

IL-8 protease (SpyCEP) is a cytokine mainly involved in the recruitment of neutrophils to a target antigen and their activation. The SpyCEP enzyme derived from the M81 strain of GAS was shown to cleave IL-8 and thereby inhibit the action of neutrophils and thereby promote bacterial migration into the host tissue. Turner and his colleagues [101] were able to induce significant systemic immunity in mice against *S. pyogenes* and *S. equi* by using a recombinant form of the N-terminal of the SpyCEP proteinase as a vaccine candidate. A study by Pandey and her colleagues [102] were able to further develop this principle by first identifying six epitopes in murine SpyCEP and testing them for their antigenic and immunogenic properties. They discovered that a specific epitope, S2, was recognized in human plasma pools also. But the antibody titers raised against these epitopes were low. However, when they combined this epitope with a DT epitope and a J8-DT epitope and injected it into the mice, a significantly better immune response was observed.

One hypothesis currently being researched is the development of vaccines using a combination of N-terminal peptides and conserved protein epitopes. This could be particularly useful in regions with high serotype diversity. Brandt et al., in 2000 [103], designed a vaccine candidate containing seven N-terminal peptides combined with an M-protein conserved region sequence (known as J14). This was assembled on a polymer bed, which served as a carrier for the vaccine. This was then injected into BALb/c mice. They were able to successfully elicit an immune response in the mice. They also tested its opsonization activity against two specific GAS strains whose c-terminal sequences were present on the J14 peptide, but the N-terminal polypeptide sequence was present only on one strain. The study showed that the two components complementarily protected against the GAS strains (Table 3).

**Table 3:** Status of clinical trials of various vaccine candidates against group A streptococcus. ^a^Signifies that the vaccine candidate is currently in that particular stage of trials.

Vaccine candidate	Pre-clinical	Phase 1	Phase 2	Phase 3
Combo5	X^a^			
Multivalent (4,6, 8 valent)	X			
26-Valent	X	X	X	
30-Valent	X	X		
J8	X	X		

Indian Scenario

In India, the search for an effective vaccine against group A streptococci is still undergoing. One of the main reasons behind it is a large amount of diversity in the strains found in India. While strains in western countries show a significant level of homogeneity, i.e., the diversity of strains in those countries is less, emm types in developing nations like India show great heterogeneity. The multivalent vaccines discussed above have been developed based on the strains found in Europe and North America. Though they show promise as vaccine candidates in those populations, their poor sequence coverage of the serotypes found in developing nations means that there is a need for a more effective vaccine that is native to the specific population.

There is very little literature available regarding vaccine research in India. There exists a vaccine for *Streptococcus pneumoniae* that is a multivalent conjugate vaccine. But this is an α-hemolytic streptococcus [104]. Potential vaccine candidates for β-hemolytic streptococci in a population as diverse as India’s are still being studied. The region-wise heterogeneity in the emm-genes makes it difficult for one to come up with an effective multivalent vaccine. A study by Abraham looked at the epidemiology and the effectiveness of GAS vaccine candidates that have been developed outside of India. According to this, only 16% of the 135 strains collected for the study were covered by the 30-valent vaccine developed by Abraham and his colleagues [12]. The emm serotypes used in the study only covered around 28% of the different strains isolated from South India. The study also made use of the emm clustering system. They found that nearly 80% of the GAS isolates could be grouped into 6-emm clusters. E3 and E6 were the most dominant clusters among them. This could help us in figuring out which M proteins or emm clusters can be used as potential candidates in any multivalent vaccine that is considered to be better suited for the Indian population. One hurdle in terms of the development of an efficient GAS vaccine in India is the high rate of mutation observed in the bacteria. Vaccines based on specific serotypes prevalent in a certain population at one time may be ineffective after a few months [105]. This was studied by Anthony and his colleagues [106]. They observed a gradual change in the prevalence of serotypes in a controlled urban population over a period of two years. This dynamic situation in the prevalence of serotypes makes it very difficult for a successful M protein-based vaccine to be developed, especially in an Indian population where the variation of serotypes observed in the population is high.

An alternative to the development of multivalent vaccines based on the amino-terminal of the M protein (which could be a difficult task considering the large number of serotypes that are prevalent in India) can be the development of vaccines that target the conserved C-repeat regions or other non-M protein-based vaccines. However, some of the vaccines based on the C-repeat regions (like the J14 vaccine) can be susceptible to sequence variance. This factor needs to be addressed and monitored carefully during the vaccine’s development process. Gupta [107] and his colleagues highlighted another issue regarding the use of C-region-based M-protein vaccines. They used J14 as the model vaccine for their experiments and found that though the J14 vaccine was able to induce immunity against multiple serotypes of the bacteria, there was still a significant risk of cross-reactivity occurring and an increasing chance of autoimmune diseases like ARF and RHD occurring. Sagar and his colleagues in 2012 [66] carried out a detailed study of the GAS genome, in which they stipulated that constructing multivalent vaccines using proteins critical to its virulence and biological function could be a promising approach to developing an effective vaccine. The existing vaccines in these categories will still need to be tested on subjects native to the Indian population first in order to determine the vaccine’s efficacy. Sharma and his colleagues [108] used various bioinformatics and proteomics-based methods to identify a panel of 147 proteins that could potentially be used as vaccine candidates. Fifty-two proteins out of these were found to be common in both sets of results. Many of these proteins had not been characterized previously. They used the M1 and M49 strains of Streptococcus for their experiments. Certain proteins, like signal peptidase 1 and laminin-binding protein (LBP protein), which mediates adhesion to the basal ganglia protein laminin, have been previously reported as potential vaccine candidates. Certain proteins were discovered whose function was known in other bacteria but not in GAS. The characterization of these new proteins could help us figure out which exact proteins can be used as potential vaccine candidates. In a follow-up study [109], Sanduja and her colleagues surveyed various cell surface proteins in order to discover a universal vaccine candidate based on various conditions.

They found a specific protein, Spy_2191, which was found in 98% of all Streptococcus genomes. This protein was found to play an important role in host cell adherence and invasion and is expressed during phagocytosis. Upon examining its immunogenic activity, it was found that it induced a significant adaptive immune response, particularly an IgG response. Significant secretion of IL-10, IL-6, and IL-17A was also observed. Overall, this vaccine candidate was shown to significantly alleviate the pharyngeal infection when the test subjects were given an intranasal dose.

Further research on the various vaccine candidates discussed above and others currently being researched will greatly help in combating what has been a resurgent pathogen over the last few decades [110].

## Conclusions

Group A streptococcal infections are becoming an increasingly prevalent health concern in today’s world, especially in economically poorer regions such as India and Southeast Asia. Over the years, many candidate vaccine molecules have been researched that are based on various parts of the streptococcal genome. These include M proteins, cell wall carbohydrates, fibronectin-related proteins, etc. M-protein vaccines have shown promising results in clinical trials mainly due to their high immunogenicity, but one of their main drawbacks is the high degree of serotype specificity, which reduces their efficacy in countries that show a higher serotype diversity. An alternative to this has been the development of vaccines based on the conserved region of the M-protein or the streptococcal genome. Advances in vaccine development have also led to the introduction of vaccines consisting of peptides from the conserved peptides from the streptococcal genome attached to the peptides found in the variable region N-terminal of the M-protein. This combines the high immunogenicity of the hypervariable M-protein region with peptides and the coverage gained by the use of peptides from the conserved regions, and thus could be the most viable option in reducing and eliminating the burden of GAS infections, especially in countries where high serotype variation is observed. This, however, requires further research on the compatibility of the different components used and also on the compatibility of the proposed delivery system.
